# Fluorodeoxyglucose positron emission tomography (18F-FDG PET)-computed tomography (CT) in the initial staging of bladder cancer: a single institution experience

**DOI:** 10.1186/s43046-023-00180-5

**Published:** 2023-07-17

**Authors:** Mohammed Shahait, Ramiz Abu-Hijlih, Ala’a Farkouh, Shahed Obeidat, Samer Salah, Ahmed Saad Abdlkadir, Akram Al-Ibraheem

**Affiliations:** 1 Surgery Department, Clemenceau Medical Center, Dubai, United Arab Emirates; 2 Radiation Oncology Department, King Hussein Cancer Center, Amman, Jordan; 3 Surgery Department, King Hussein Cancer Center, Amman, Jordan; 4 Nuclear Medicine Department, King Hussein Cancer Center (KHCC), P.O. Box 1269, Al-Jubeiha, Amman, 11941 Jordan; 5 Medical Oncology Department, King Hussein Cancer Center, Amman, Jordan; 6 School of Medicine, University of Jordan, Amman, Jordan

**Keywords:** Bladder cancer, PET scan, Staging, Conventional imaging

## Abstract

**Background:**

The purpose of this study was to assess the usefulness of fluorodeoxyglucose positron emission tomography (^18^F-FDG PET)-computed tomography (CT) scan for staging urinary bladder cancer. The study also sought to determine the effect of ^18^F-FDG PET/CT on management decisions and its implications for patient care.

**Methods:**

A total of 133 patients with bladder cancer who had both conventional imaging and ^18^F-FDG PET/CT for initial staging were identified. All ^18^F-FDG-PET/CT findings were classified as true positive, true negative, false positive, or false negative based on their potential to impact the intent of treatment. The sensitivity, specificity, positive predictive value, and negative predictive value were calculated using the standard definition. Furthermore, the rate of change in therapy intent was determined for the entire sample and for subgroups with non-muscle-invasive bladder cancer (NMIBC) and muscle-invasive bladder cancer (MIBC) patients.

**Results:**

The overall concordance rate between PET/CT and conventional imaging was around 54%. On conventional images, 18% of patients had localized disease, which was upstaged in 6.8% of cases using ^18^F-FDG PET/CT. Pelvic lymph node involvement was detected in 18.8% of cases using conventional imaging, which was downstaged to localized disease in 4.5% of cases using ^18^F-FDG PET/CT. While 63.2% of patients had systemic disease on a CT scan, 24.7% of cases were downstaged using PET/CT. Overall, the rate of change in therapy intent was 26.3% for the entire sample, 24.5% for NMIBC subgroup, and 27.3% for MIBC patients.

**Conclusions:**

The study found that ^18^F-FDG PET/CT is an effective and accurate tool for staging bladder cancer in newly diagnosed patients. Approximately one quarter of patients had a change in management intent based on ^18^F-FDG PET/CT results. The study suggests that PET/CT should be used as a standard for newly diagnosed patients, but more research is needed to confirm this.

## Background

Bladder cancer is one of the most common malignancies worldwide, ranking tenth among cancers with a prevalence of almost 1.65 million worldwide and an incidence of over half a million new cases each year across the globe. These numbers are expected to increase in the future, mainly due to the increasing population size as well as aging [1]. In the USA alone, more than 80,000 new cases of bladder cancer are diagnosed every year [2].

The most common sub-type of bladder cancer is urothelial carcinoma, accounting for more than 90% of cases, with many histological variants that have been identified and classified by the World Health Organization [3].

Broadly, bladder cancer can be classified into NMIBC which accounts for almost 70% of cases and MIBC which represents the remaining 30% [4]. The diagnostic work-up and treatment strategies vary between the two subgroups. For NMIBC, imaging for distant metastasis is not routinely performed, and the mainstay treatment includes local treatments with transurethral resection of the tumor, with adjunct intravesical chemotherapy or Bacillus Calmette-Guerin (BCG) depending on the risk stratification of the affected patient [5, 6].

On the other hand, the staging work-up of MIBC is crucial to detecting metastatic lesions. Management of MIBC is dictated by stage of the disease. Metastatic MIBC is treated with systemic chemotherapy or immunotherapy [6], while for non-metastatic MIBC (T2-T4, N0, M0), the standard recommended treatment is neoadjuvant cisplatin-based chemotherapy followed by radical surgery or concurrent chemoradiation with curative intent [7, 8]. CT scan with intravenous (IV) contrast of the chest, abdomen, and pelvis is the standard imaging modality for staging that can assess the disease locally, in addition to detecting lymph node, lung, and visceral metastases. Furthermore, it can detect upper urinary tract involvement [8, 9]. In MIBC, 25% of patients with a normal CT scan prior to cystectomy are found to have lymph node metastases, the majority of which are microscopic metastases [10]. This might be attributed to the inherent limitations of the low resolution of CT scan images and the variation of MRI protocols used to stage bladder cancer patients [11]. Moreover, the prevalence of pre-operative renal insufficiency in patients with bladder cancer was estimated at 16.9% (ranging from 13.0 to 25.5%), which might preclude the use of conventional images in a substantial number of patients [12]. Over the last decade, there has been heightened interest in examining the role of ^18^F-FDG-PET/CT in staging bladder cancer staging. Interestingly, some studies found that the management plan for approximately 68% of patients can be changed based on the ^18^F-FDG-PET/CT results [13].

Although body of evidence suggests that PET/CT scan has a higher sensitivity for detecting lymph node metastasis compared to conventional CT scan, the American Urological Association (AUA) and European Association of Urology (EAU) guidelines do acknowledge its clinical use but do not recommend it as the initial staging modality [7, 8]. EAU did not make any particular recommendations [8], while the AUA recommends its use if conventional chest, abdomen, or pelvic images reveal findings which need further evaluation or if biopsy of a suspicious lymph node is not possible [7]; this in particular is related to the factor that data on the use of a PET/CT for initial staging is scarce. Moreover, the intricate relationship between genomic makeup of the tumor and radiomics features obtained from PET-Scan is an area of current investigation [14]. Giving the bladder cancer tumor genomic heterogeneity among different ancestries, we sought to evaluate the use of a PET/CT scan for initial staging of bladder cancer in patients with Middle Eastern ancestry and the implications this carries in terms of management at a tertiary cancer center.

## Methodology

### Patient population

After obtaining the Institutional Review Board’s approval and waived patients consent, a retrospective chart review was done for bladder cancer patients who underwent ^18^F-FDG PET/CT for initial staging at King Hussein Cancer Center, between June 2018 and December 2020. All included patients had a tissue diagnosis of bladder cancer reviewed at our institution by a genito-urinary (GU) specialized pathologist. All patient’s management plan were based on a discussion at the GU multidisciplinary team consisted of urologists, radiation oncologists, pathologists, radiologists, nuclear medicine physician, and medical oncologists. The decision usually is based on patient age, comorbidities, performance status, and pathology.

Patients’ demographics, tumor stage, histology, and subsequent treatment were collected and recorded. Patients with no baseline imaging using CT/magnetic resonance imaging (MRI) were excluded. The findings of ^18^F-FDG-PET/CT were compared with anatomic imaging to evaluate the effect on disease stage.

### Staging imaging

The clinical standard for initial staging of bladder cancer includes a CT scan of the chest, abdomen, and pelvis. Accordingly, all patients included in this study underwent conventional imaging as part of their initial staging. At the time of diagnosis, a contrast-enhanced CT scan of the neck, chest, abdomen, and pelvis had been performed on all patients. Additionally, periodic follow-up examinations were performed. The CT findings were analyzed in terms of the main affected area, the extent of involvement in that area, the presence of additional affected areas, and any complications.

On the other hand, ^18^F-FDG PET/CT was not routinely performed in all patients but only to further evaluate indeterminant findings on conventional imaging (e.g., borderline lymph node, suspicious bone/lung lesion). Patients were scheduled 4–6 weeks after bladder biopsy to minimize the risk of false positive results. The PET-CT images were acquired on the Biograph mCT Flow PET/CT scanner (Siemens Medical Solutions, Erlangen Germany). The patients were injected with the ^18^F-FDG (3 MBq/per kg, minimum of 125 MBq) by IV injection after at least 6-h fasting and blood glucose level below 200 mg/dl. Imaging acquisition was carried out from vertex to mid thighs (standard Protocol), 60–90 min after the injection. PET images were obtained through the use of FlowMotion technology in a 3D mode. The acquisition process employed a table speed of 1 mm/s, corresponding to a duration of 3 min per bed position. Images were taken after the administration of IV fluids and a diuretic to reduce bladder activity [15]. The aforementioned approach is known to facilitate primary tumor detection through reducing urine retentions [10]. It is also helpful in improving urine output that is necessary to washout any background urine metabolic activity [10]. Therefore, all patients were subjected to aforementioned approach. The scans were assessed by dedicated and experienced nuclear medicine physicians. The maximal standardized uptake value (SUVmax) was determined by taking into account the applied activity, administration time, and patient weight. The SUVmax value was calculated for each tumor focus that had high FDG uptake [16]. The main criterion of positivity was the presence of focal uptake of the ^18^F-FDG in one or more locations, higher than in the surrounding tissue background, excluding the articular processes and physiological uptake areas (e.g., excreted urine activity in the ureters and adnexal activity). The findings of conventional imaging and the ^18^F-FDG PET-scan were presented at our multidisciplinary clinic, where the results were correlated with patient history, clinical examination, and anatomical images. The final treatment plan was based on panel recommendations.

### Reference standard

All ^18^F-FDG-PET/CT findings were classified as true positive, true negative, false positive, or false negative based on the clinically relevant stage that would impact the intent of treatment (local disease, pelvic lymph nodes only regardless of laterality, and distant metastasis including visceral and bone). As described previously by Apolo et al., any lesion that was detected on PET/CT and confirmed to be malignant by serial conventional images or response to systematic treatment was considered true positive [13]. A finding was considered true negative if it was not detected on PET/CT and subsequent serial conventional axial imaging did not show evidence for malignancy, such as an increase in size or no response to chemotherapy [13]. A lesion seen on the initial PET/CT was considered a false positive if suspicious ^18^F-FDG uptake was described on the ^18^F-FDG PET/CT, but the subsequent serial imaging studies favored benign processes, such as no increase in size or no response to chemotherapy. A false-negative lesion is defined as a lesion that is not detected by PET/CT but is initially seen on conventional images, and subsequent imaging studies show morphological progression or response to systemic treatment [13]. In the event of a discordant finding, a true-positive lesion takes precedence over all other lesions, including false negatives, true negatives, and false positives [13]. As such, if a patient has at least one true-positive lesion (whether it is primary, nodal or metastatic), the PET/CT scan is considered true-positive (for its correlated site of origin whether primary nodal or metastatic) [13]. In the absence of a true-positive lesion, a false-negative lesion takes precedence over a true-negative or a false-positive lesion [13]. As a result, if the PET/CT is false negative in at least one disease site (within primary, nodal or metastatic regions), it is regarded as a false negative overall ((within primary, nodal or metastatic regions) [13].

### Statistical analysis

Descriptive statistics were utilized when appropriate to report means, median, standard deviations, and proportions. The sensitivity, specificity, positive predictive value, and negative predictive value were all calculated using the standard definitions. All statistical analyses were performed using SPSS version 19 (SPSS Inc., Chicago, IL).

## Results

Out of 420 patients with bladder cancer who completed their initial evaluation between June 2018 and December 2020, 137 (32.6%) had an ^18^F-FDG PET/CT scan. Four patients with no baseline anatomical imaging were excluded. Among all 133 patients included, the median age at staging was 65 years (57–73). Forty-nine (36.9%) patients had NMIBC, 84 (63.1%) patients had MIBC, 35 (26.3%) patients had pelvic lymph nodes only, and 69 (51.9%) patients had metastatic disease (Table 1).

**Table 1:** Demographics and clinical features of included sample

**Age (in years)**	
Median	65 years
Range	57–73 years
**Gender (*n*, %)**	
Male	113, 85%
Female	20, 15%
**Tumor extension (*n*, %)**	
Confined locally	29, 21.8%
Nodal involvement	35, 26.3%
Metastatic	69, 51.9%
**Muscle invasion**	
Negative	49, 36.9%
Positive	84, 63.1%
**Surgical management (after staging)**	
Cystectomy	44, 33%
Transurethral resection of bladder tumor (TURBT)	89, 67%

**Fig. 1:**
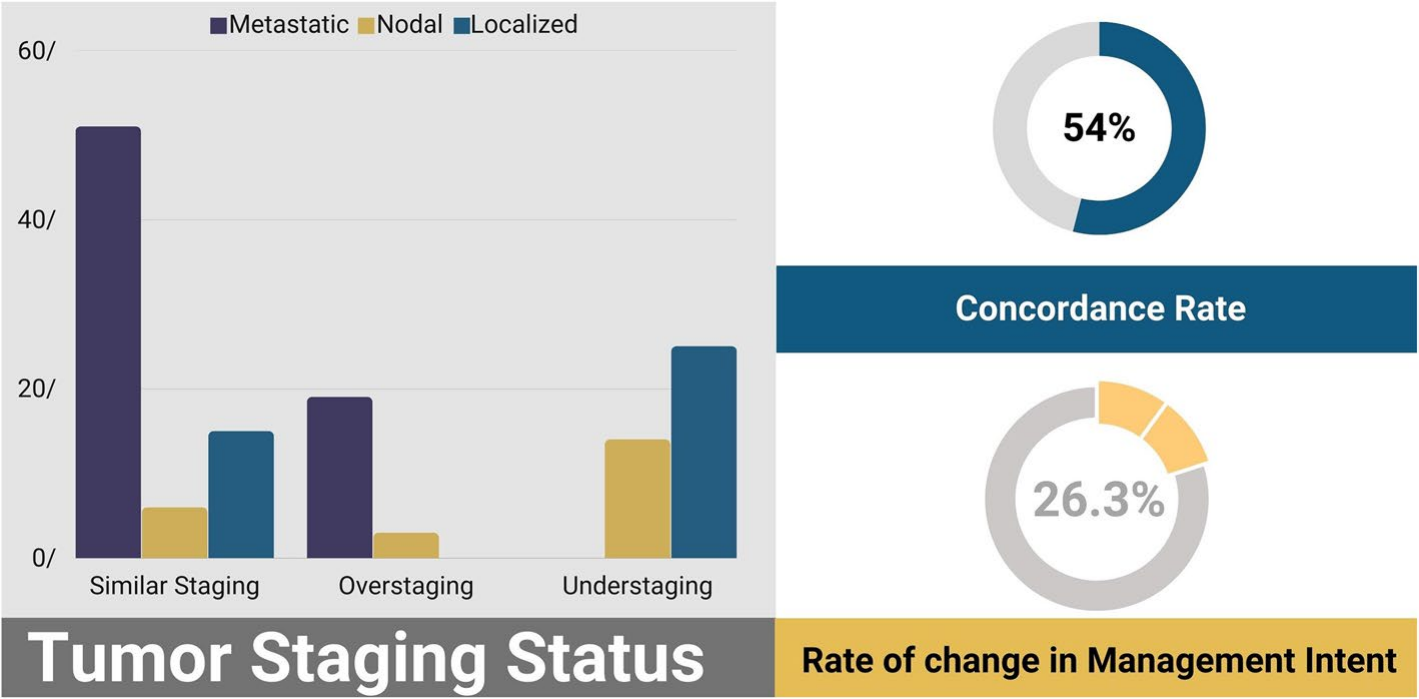
Overall concordance rate and patterns of change in Staging and management achieved through PET/CT compared against whole-body CT

**Fig. 2:**
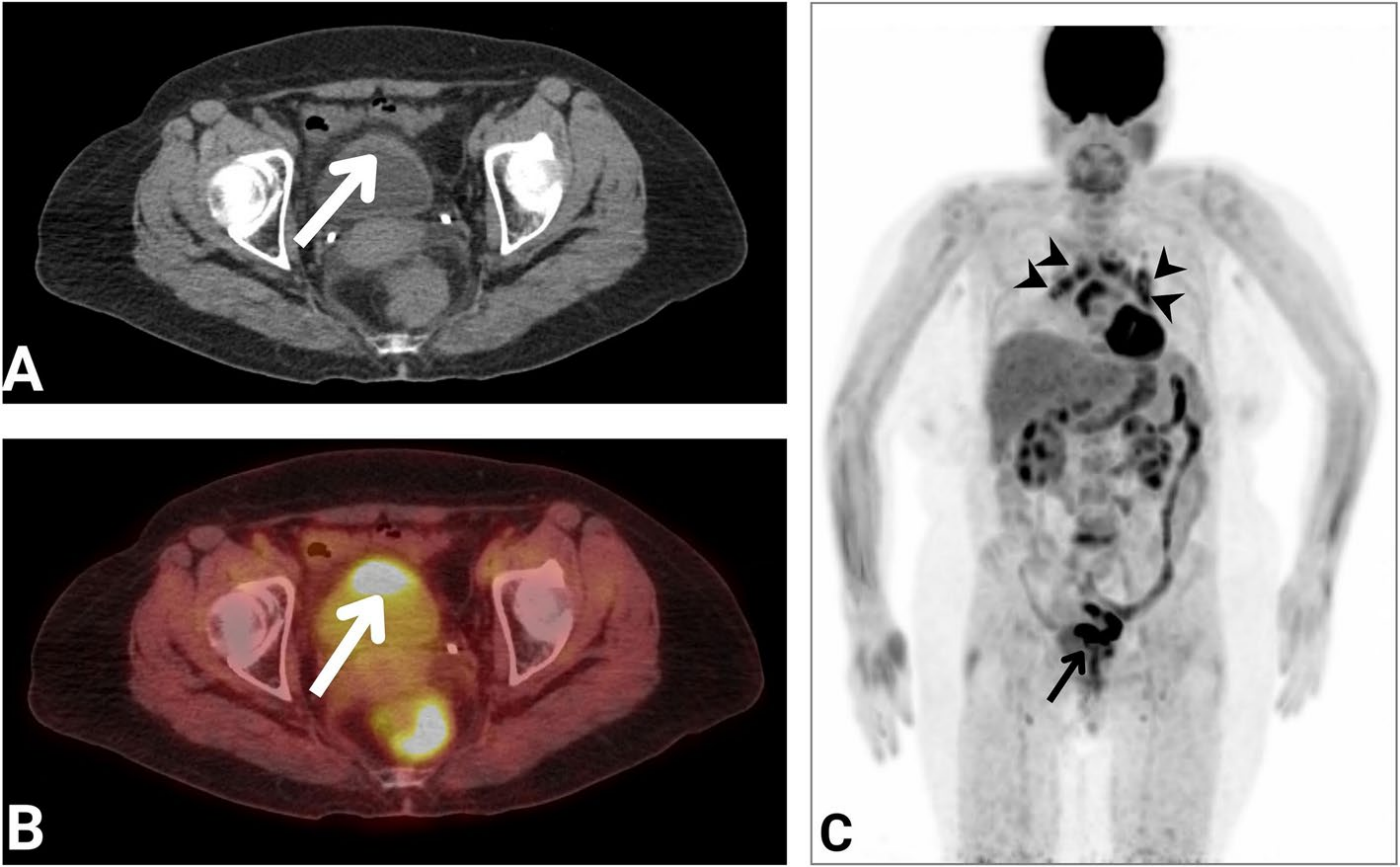
A 69-year-old patient diagnosed with bladder cancer who underwent ^18^F-FDG PET/CT for staging. Axial CT scan (**a**) revealed anterior bladder wall thickening (white arrow). Axial fused PET/CT images (**b**) demonstrated intensely hypermetabolic anterior bladder wall thickening (white arrow). The MIP PET scan (**c**) showed the FDG focus within the urinary bladder (black arrows). Additionally, there was ancillary evidence of bilateral symmetrical hilar lymphadenopathy. This was initially reported as granulomatous disease, which was later validated (black arrow heads)

**Fig. 3:**
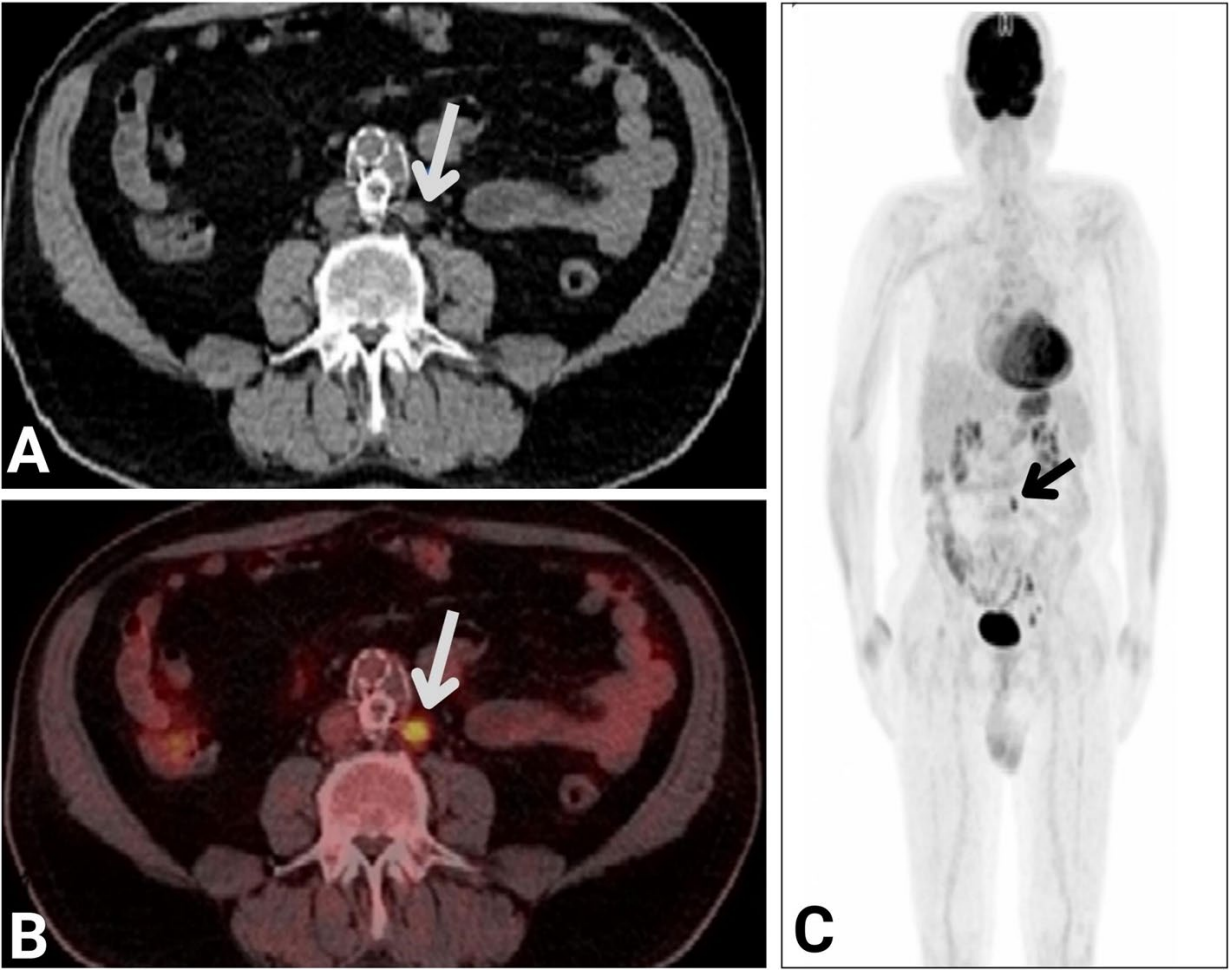
A 73-year-old patient diagnosed with bladder cancer who underwent ^18^F-FDG PET/CT for staging. Axial CT scan (**a**) showed 6 mm retroperitoneal lymph nodes (white arrow) that was not considered suspicious based on CT scan morphologic criteria. Axial fused PET/CT images (**b**) demonstrated the hypermetabolic retroperitoneal features of this small lymph node, denoting its metastatic nature (white arrow). MIP PET (**c**) shows the hypermetabolic abdominopelvic lymph nodes (black arrow)

**Fig. 4:**
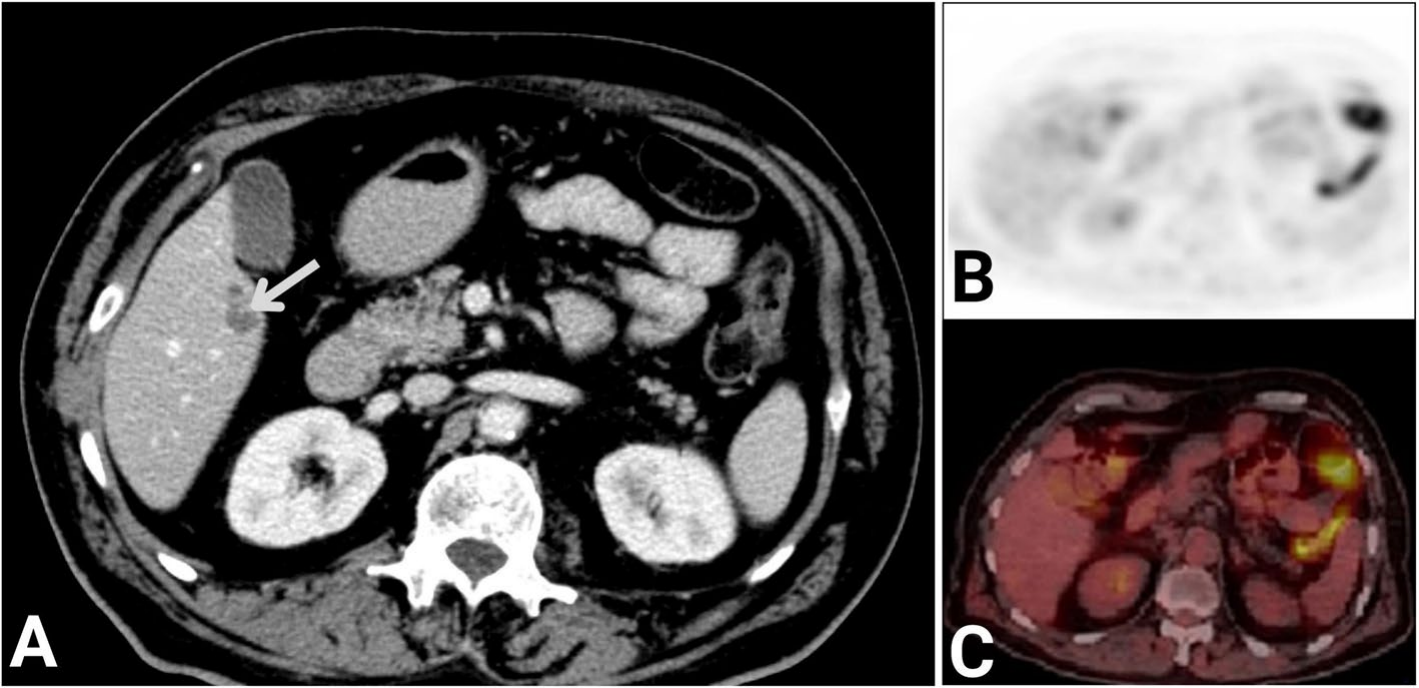
A 60-year-old patient diagnosed with bladder cancer who underwent ^18^F-FDG PET/CT for staging. Diagnostic contrasted CT (**a**) showed a suspicious liver lesion in segment V (white arrow) that was referred for further evaluation. Axial PET (**b**) and axial PET/CT images (**c**) did not demonstrate any increased metabolic activity in this lesion suggesting being benign in nature. This which was later proven on follow-up

The overall concordance rate between the two modalities was 54% (72/133) (Fig. 1). On conventional images, 18% (24/133) of the patients had localized disease (Fig. 2), upstaged using PET/CT in 9/133 cases (Fig. 3). Pelvic lymph node involvement was detected in 18.8% (25/133) of cases using anatomic imaging, downstaged using PET CT in 4.5% (6/133) for localized disease, and upstaged in 9.8% (13/133) for distant disease. While 63.2% (84/133) of patients had systematic disease on CT scan, 24.7% (33/133) were downstaged to localized disease in 14.2% (*N* = 19) and only pelvic lymph node disease in 10.5% (*N* = 14) on PET CT (Fig. 4). Management was discussed at the multidisciplinary level; the intent of treatment was changed from curative to palliative in 12% (16/133) of the patients. In 14.3% (19/133) of cases, the intention shifted from palliative to curative treatment (Table 2). Table 2 summarizes the distribution of lesions detected by each imaging modality. Overall, the rate of change in therapy intent was 26.3% for the entire sample, 24.5% for NMIBC subgroup, and 27.3% for MIBC patients.

**Table 2:** Table summarizing the distribution of lesions detected by initial imaging modalities

**Modality**	**Extent**	**Patients**	**Percentage**
**CT scan**	**Localized**	24	18%
	**Nodal**	25	18.8%
	**Metastatic**	84	63.2%
**PET/CT scan**	**Localized**	40	30%
	**Nodal**	23	17%
	**Metastatic**	70	53%
**Mutual**	**Localized**	15	11.1%
	**Nodal**	6	4.5%
	**Metastatic**	51	38.4%
**Overall concordance**	**Patients:** 72		54%

### Clinical stage-based analysis

As above-mentioned, patients had evaluable PET/CT scans that were followed by a follow-up conventional scan. The sensitivity and specificity of the PET/CT to detect pelvic lymph nodes were 54.3% (95% confidence interval (CI): 36–71%) and 98.9% (95% CI: 94–99%) in those with a follow-up scan, respectively. Based on these findings, the positive predictive value is 96.2%, and the negative predictive value is 82.2% (Table 3). While the sensitivity and specificity of the PET/CT to detect metastasis were 76.8% (95% CI: 65–86%) and 96.9% (95% CI: 89–-99%) in those with a follow-up scan, respectively (Table 3).

**Table 3:** Table demonstrating diagnostic performance of FDG PET/CT scan compared to a reference standard obtained from follow-up imaging and final clinical diagnosis

Disease extension	TP	FN	TN	FP	Sensitivity (conf int)	Specificity (conf int)	PPV (conf int)	NPV (conf int)	Accuracy (conf int)
Confined locally	15	14	100	4	51.7% (32–70%)	96.2% (90–99%)	79.1% (58–91%)	87.6% (83–91%)	86.4% (79–92%)
Pelvic lymph node involvement	19	16	97	1	54.3% (36–71%)	98.9% (94–99%)	96.2% (77–99%)	82.2% (76–87%)	84.7% (77–90%)
Metastatic disease	53	16	62	2	76.8% (65–86%)	96.9 (89–99%)	96.4% (87–99%)	79.4% (71–86%)	86.4% (79–92%)

## Discussion

In urinary bladder carcinoma, CT, FDG-PET/CT, and MRI are used for the radiological diagnosis and follow-up of bladder cancer. FDG-PET/CT is more effective in detecting lymph nodes and distant organ metastasis, while MRI is better at performing T-staging or evaluating local disease [16–19]. CT has moderate accuracy in bladder carcinoma, but its ability to detect local staging decreases with extravesical invasion of the tumor and microscopic metastases in regional and distant lymph nodes [20–22]. MRI is more accurate than CT in the local staging of bladder cancer, correctly distinguishing superficial from invasive tumors and organ-confined from extensive disease 85% and 82% of the time, respectively [16]. However, the use of FDG-PET/CT in bladder cancer is still an area of research. The presence of high background FDG activity in urine limits its ability to evaluate the local spread of the disease and regional metastases. This has led to the development of some methods for using FDG-PET/CT for local assessment, such as intravenous furosemide injection followed by forced diuresis and additional images from the pelvic region taken 1 h later. Using this technique, Nayak et al. have found that the sensitivity of FDG-PET/CT in local assessment and locoregional lymph nodes is higher than that of contrast CT [23]. In a similar study, Harkirat et al. found that 16 out of 29 patients had positive foci in the tumor region in the late diuretic images [24]. Furthermore, 7 of these 16 lesions could only be detected with PET and not with CT [24]. Anjos et al. also observed upstaging using late diuretic imaging [25]. This method was successful in improving the detection of locally recurrent or residual bladder cancer [25].

In this study, we found that the PET-CT scan used at the initial staging for bladder cancer changed the intent of treatment in 26% of the patients. The overall concordance rate between the two modalities was 54% (72/133).

Few studies have looked at the use of the ^18^F-FDG-PET/CT scan in bladder cancer, whether for initial staging, restaging, or assessing response to therapy [26]. The sensitivity of the PET/CT to detect pelvic lymph nodes in this study was 54.3% (95% CI: 36–71%), which is in line with that reported in the literature [27, 28]. In a meta-analysis comprising 8 studies, the pooled sensitivity for detecting lymph node metastasis in MIBC using PET/CT was 57%, compared to the pooled sensitivity of 35% when using a conventional CT scan [27]. On the other hand, the sensitivity of the PET/CT in our cohort to detect metastasis was 76.8% (95% CI: 65–86%), which is comparable to the ^18^F-FDG PET/CT sensitivity ranges between 54 and 87%, reported in the literature [29].

The utility of the PET scan in staging bladder cancer emerges from its impact on clinical management intent. Apolo et al. found that 68% of patients with bladder cancer had their treatment changed based on the findings of the ^18^F-FDG PET/CT. However, the treatment intent was changed for only 26% of the cohort [13]. Indeed, this is in concordance with the results from our cohort, which showed that 26% (35/133) of patients’ treatment intent was changed from curative to palliative, or vice versa. In urinary bladder cancer, primary tumor metabolic assessment through the SUVmax value is quite a difficult task due to the high background activity of urine. Nonetheless, many of the previous studies have found that tumors with high SUVmax values represent an aggressive pattern, may be correlated with a high risk of recurrence, and may impact survival outcome [30]. More recently, Novruzov et al. evaluated the potential of ^68^Ga FAPI-PET/CT in comparison to ^18^F-FDG PET/CT for assessment of bladder cancer in patients with untreated urinary bladder cancer [31]. Results showed that ^68^Ga FAPI demonstrated significantly higher uptake in both the primary tumor and metastases when compared to ^18^F FDG PET/CT [31]. Furthermore, ^68^Ga FAPI was able to detect an additional 30% of lesions that had been missed by ^18^F FDG [31]. Based on these findings, the study concludes that ^68^Ga FAPI-PET/CT is a more effective method for detecting metastatic lesions in patients with advanced bladder cancer and has great potential as a theranostic agent for urological cancer diseases [31]. Significant progress in the areas of nuclear oncology and molecular imaging can be achieved through the application of advanced whole-body protocols utilizing digital PET and whole-body PET scanners [32]. These cutting-edge devices offer not only rapid imaging protocols but also increased sensitivity improved spatial and temporal resolution, enhanced signal-to-noise ratio, and decreased radiation exposure, which contribute to more precise imaging results across various types of cancer [32].

Despite the evolution of the treatment of bladder cancer with the introduction of novel agents such as immunotherapy, FGFR3 inhibitors and the embrace of trimodal therapy (TMT) for localized disease [33]. Bladder cancer management in locally advanced as well as metastatic setting is challenging [34]. For example, patients with pelvic lymph node metastasis are not candidates for TMT, are less likely to benefit from radical cystectomy, and might benefit from early systematic treatment intensification.

Our study suffers from its retrospective nature and associated limitations. Moreover, the clinical impact was assessed by medical record review, not by a prospective questionnaire. Nevertheless, it represents a large series of bladder cancers, managed at a multidisciplinary level. Our results add to the current body of evidence and support the use of PET/CT at initial presentation.

## Conclusions

Our study demonstrates the efficacy and diagnostic advantage that PET/CT offers for staging in patients newly diagnosed with bladder cancer. In approximately one quarter of patients, the management approach and intent to treat have significantly changed. A larger prospective study and a cost-effectiveness analysis are still warranted to support the use of PET/CT as a standard for newly diagnosed patients.

## Data Availability

The data is available upon reviewer’s/editor’s request to the corresponding author.

## Abbreviations

^18^F-FDG PET: ^18^Fluorodeoxyglucose positron emission tomography
CT: Computed tomography
NMIBC: Non-muscle-invasive bladder cancer
MIBC: Muscle-invasive bladder cancer
BCG: Bacillus Calmette-Guerin
IV: Intravenous
AUA: American Urological Association
EAU: European Association of Urology
GU: Genito-urinary
MRI: Magnetic resonance imaging
SUVmax: Maximal standardized uptake value
CI: Confidence interval
TMT: Trimodal therapy
